# Professional quality of life, sleep quality, occupational stress, and turnover intention among emergency department nurses: A cross-sectional study

**DOI:** 10.1371/journal.pone.0357570

**Published:** 2026-09-22

**Authors:** Sogand Sarmadi, Neda Sanaie, Fariba Borhani, Akbar Zare-Kaseb, Ali Zeidabadi, Sara Beigzadeh, Haniyeh Ghanbari Nasab

**Affiliations:** 1 Medical Ethics and Law Research Center, Shahid Beheshti University of Medical Sciences, Tehran, Iran; 2 Department of Medical-Surgical Nursing, School of Nursing and Midwifery, Shahid Beheshti University of Medical Sciences, Tehran, Iran; The Affiliated Changzhou No 2 People's Hospital of Nanjing Medical University, CHINA

## Abstract

**Background:**

Emergency department (ED) nurses face a heavy workload and high psychological demands that may adversely affect their professional quality of life (Pro-QoL), sleep patterns, occupational stress, and turnover intention. This study examined the associations between ProQoL, sleep quality, occupational stress, and turnover intention among ED nurses, and identified factors associated with turnover intention.

**Methods:**

The study, conducted in 2025, employed a descriptive cross-sectional design and included 180 nurses in the ED of university hospitals in Tehran Province. Data were gathered using the Professional Quality of Life Scale (ProQOL), the Pittsburgh Sleep Quality Index (PSQI), the Expanded Nursing Stress Scale (ENSS), and the Turnover Intention Questionnaire. Descriptive statistics, Spearman rank correlation, Mann-Whitney U and Kruskal-Wallis H tests, and robust regression were employed in the data analysis.

**Results:**

The mean score of turnover intention was 49.26. Turnover intention was significantly associated with sleep quality (r = 0.329), job stress (r = 0.408), burnout (r = 0.575), compassion satisfaction (r = −0.555), and secondary traumatic stress (STS) (r = 0.594) (p < 0.01). In the regression analysis, compassion satisfaction, burnout, STS, job stress, financial status, nurse-to-patient ratio, and shift type were significant predictors of turnover intention.

**Conclusion:**

These findings suggest that Pro-QoL, occupational stress, financial status, shift scheduling, and nurse-to-patient ratio are significantly associated with turnover intention among ED nurses. Longitudinal research is needed to establish causal pathways and evaluate targeted interventions.

## 1. Introduction

Emergency department (ED) nurses are exposed to uniquely demanding working conditions, including high patient acuity, unpredictability, traumatic presentations, and resource constraints, that place them at elevated risk of compromised professional well-being compared with nurses in general wards [1,2].

Professional Quality of Life (Pro-QoL) refers to the quality one experiences in relation to helping others who are suffering or have experienced traumatic events. According to the Pro-QoL framework, Pro-QoL consists of both positive and negative dimensions. The positive dimension is Compassion Satisfaction (CS), which reflects the pleasure and fulfillment derived from performing one’s work effectively, helping others, and contributing to the well-being of individuals and society. The negative dimension is Compassion Fatigue (CF), which comprises two related but distinct components: Burnout and Secondary Traumatic Stress (STS). Burnout is characterized by feelings of hopelessness, frustration, exhaustion, and difficulties in performing work effectively, often developing gradually in response to high workload or an unsupportive work environment. In contrast, STS is a negative, fear-related reactions arising from indirect exposure to traumatic events, that is, from caring for, supporting, or being repeatedly exposed to the suffering of traumatized patients, rather than from directly experiencing the event oneself. Thus, ProQOL reflects the balance between the rewarding and distressing aspects of helping professions [1,3–6]. Several investigations have documented moderate to high levels of STS and burnout among ED nurses. In one study, the average levels of burnout and STS, defined as ProQOL raw subscale scores of 23–41 according to the Pro-QoL manual, were reported to affect 91.4% and 85.2% of this population, respectively [3].

Prior investigations have established an inverse relationship between sleep disturbances and professional outcomes, including job satisfaction and overall well-being, within the nursing profession. A significant proportion of nurses report insufficient sleep or excessive daytime sleepiness, reflecting the restorative role of sleep in physical and cognitive functioning [1]. This issue is more prevalent among shift workers. The circadian rhythms of nurses are disrupted by shift rotation and extended work hours [7]. Poor sleep quality is highly prevalent among ED nurses. A large-scale study on ED nurses in public hospitals in China reported that the prevalence of poor sleep (Pittsburgh Sleep Quality Index (PSQI)> 5) and severe sleep problems (PSQI >  8) among ED nurses was 76.3 and 68.8%, respectively [8]. Similarly, a cross-sectional study among ED nurses during the COVID-19 pandemic found that 81.5% reported poor sleep quality, as assessed by the PSQI, with an overall mean score of 10.55 [9]. Furthermore, among female ED nurses in Iran, the prevalence of poor sleep quality was reported to be 82.7% [10]. Consequently, diminished sleep quality and duration may lead to complications, including professional burnout. Notably, burnout and sleep disorders demonstrate a bidirectional influence [1].

The probability of an employee's departure, whether voluntary or via transfer, is correlated with their stated intent to leave, a decision often influenced by negative workplace dynamics, limited career progression, or other relevant circumstances [11]. A recent meta-analysis reported that the pooled turnover rate among nurses was 15.2%, and the pooled prevalence of turnover intention was 38.4%. Moreover, night-shift nurses exhibited a turnover rate almost twice that of day-shift nurses and were three times more likely to consider leaving their positions [12]. Evidence from another meta-analysis indicates that the turnover intention among younger nurses (61%) was significantly greater than that among older nurses (30%) [13]. The findings indicate that nurses’ intention to leave their institutions is strongly shaped by a combination of socioeconomic and professional factors. Lower educational status was associated with a significantly higher likelihood of turnover intention. A monthly income below 3145 Birr markedly increased the probability of intending to leave, highlighting the critical role of financial dissatisfaction and economic pressure in staffing instability. In contrast, greater professional autonomy demonstrated a protective effect against turnover intention [14].

Despite the critical role of ED nursing in healthcare delivery, the workforce continues to be affected by high turnover driven by multiple professional and organizational stressors. Identifying modifiable predictors of turnover intention is therefore essential for informing management strategies. To date, few studies have concurrently examined Pro-QoL, sleep quality, occupational stress, and turnover intention within a single sample of ED nurses, particularly in the Iranian context; the present study was designed to address this gap. This study aimed to examine the associations between Pro-QoL, sleep quality, occupational stress, and turnover intention among ED nurses, and to identify factors associated with turnover intention.

## 2. Method

### 2.1. Design

A descriptive cross-sectional design was used to conduct this study, which was reported following STROBE (Strengthening the Reporting of Observational Studies in Epidemiology) guidelines [15].

### 2.2 Participants and settings

ED nurses from teaching hospitals in Tehran Province, Iran, took part in this study from 12 March 2025 to 31 May 2025. Data were collected in the EDs of six hospitals affiliated with this university, which together employed approximately 210 ED nurses during the study period. A convenience sampling strategy was employed. To ensure adequate familiarity with the work environment, candidates were required to hold at least a bachelor's degree, be employed full-time in a clinical setting, and possess a minimum of one year of experience in ED wards. Willingness to participate was a prerequisite for nurses. To mitigate participant dropout, questionnaires were meticulously designed for ease of completion, and respondents were strongly encouraged to provide comprehensive responses. Because all items were mandatory, participants who withdrew by closing the browser window before completing all items were not included in the final dataset.

### 2.3. Sample size

Sample size was estimated based on the correlation between quality of work life dimensions and intention to leave the profession reported by Lee et al. [16]. Using Fisher's z-transformation for correlation-based power analysis (two-tailed α = 0.05, power = 90%, r = 0.25), a minimum sample of 164 participants was required. Accounting for a 10% dropout rate, the target sample was set at 180 participants. Given that the final regression model (Model 2) included up to 14 predictor variables, the achieved sample of 180 participants yields approximately 12.8 observations per predictor, which approaches but slightly exceeds conservative guidelines of ≥10 observations per variable.

### 2.4. Measures

#### 2.4.1 The turnover intention questionnaire.

Kim et al. [14] developed the turnover intention questionnaire to evaluate employees’ intent to leave their positions. A fifteen-item questionnaire gauged employee motivation and propensity to leave their current position. Leaving one's place of employment encompasses more than simply departing the physical premises. Despite physical workplace attendance, a lack of intellectual and spiritual engagement is observed among some employees. This questionnaire seeks to explore the motivations behind employee turnover. Participants rated each question using a five-point Likert scale, ranging from complete disagreement to complete agreement. The 5-point Likert scale employs the following scoring system: 5 represents complete agreement, 4 represents agreement, 3 represents neutrality, 2 represents disagreement, and 1 represents complete disagreement. The total questionnaire score was computed by summing the individual question scores. This score ranges from 15 to 75, with scores positively correlated to the probability of respondent attrition. The Persian adaptation of the questionnaire underwent validation in Iran, yielding a Cronbach's alpha coefficient of 0.82 [17]. Mirzaei et al. [18] reported a Cronbach's alpha coefficient of 0.76 for their turnover intention questionnaire. The questionnaire's validity was confirmed by expert evaluation in the study by Heidari et al. [19], and its reliability was established through a Cronbach's alpha of 0.93. A Cronbach's alpha of 0.90 was obtained for the Turnover Intention Questionnaire, signifying high internal consistency within the present study. Because no validated categorical cut-off is defined for this questionnaire, the total score was analysed as a continuous variable, with higher values indicating a stronger intention to leave.

#### 2.4.2 The professional quality of life scale (ProQOL).

This scale is among the most frequently utilized metrics for assessing the beneficial and detrimental consequences of interacting with individuals who have endured highly stressful events. The instrument, initially termed the Compassion Fatigue Self-Test, was developed by Charles Figley in the late 1980s. A questionnaire comprising thirty questions was used in this study. The questionnaire is composed of three subscales: compassion satisfaction (10 items), burnout (10 items, with reverse scoring applied to select items), and STS (10 items). The questionnaire employs a 5-point Likert scale for responses, with 1 representing “never” and 5 representing “often”. The subscales are considered independent entities; therefore, their scores cannot be combined. The validity of the questionnaire was examined and confirmed by Gorji et al. (2017). Additionally, the reliability assessment involved the calculation of the internal correlation coefficient (Cronbach's alpha), which resulted in a score of 80% [20]. In the current study, the compassion satisfaction, burnout, and STS subscales showed acceptable reliability with Cronbach's alpha coefficient of 0.8, 0.785, and 0.883, respectively. Each subscale comprises 10 items and is summed to a raw score ranging from 10 to 50, with higher scores reflecting higher levels of the corresponding construct. Following the ProQOL manual [6], subscale raw scores were interpreted as low (≤22), moderate (23–41), or high (≥42).

#### 2.4.3 Expanded nursing stress scale (ENSS).

Gary-Toft and Anderson designed and developed the Nursing Stress Scale (NSS) (34-item) in 1981 to measure the frequency and major sources of nurses’ job stress. The Expanded Nursing Stress Scale (ENSS) is an updated and extended version of the classic NSS designed to assess job-related stress among nurses in physical, psychological, and social work environments. This questionnaire has 57 questions and nine components: mortality, conflict with physicians, inadequate emotional preparation, problems with colleagues, problems with supervisors and high workload, ambiguity about time, patients and their families, and discrimination. It measures job stress based on a Likert scale. To calculate the overall score of the questionnaire, the score of all questionnaire items is added together. The range of scores for this questionnaire is between 57 and 285. The higher the score obtained from this questionnaire, the greater the level of job stress for nurses and vice versa [21]. In the study of Olyaie et al. (2010), this questionnaire's content, face, and criterion validity were assessed as appropriate. The Cronbach's alpha coefficient calculated in the study of Olyaie Khachik et al. (2010) for this questionnaire was estimated to be above 0.7 [22]. This questionnaire showed good internal consistency in the current study with a Cronbach's alpha coefficient of 0.975.

#### 2.4.4 Pittsburgh sleep quality questionnaire.

The Pittsburgh Sleep Quality Index (PSQI) assesses subjective sleep quality over the preceding month [23]. The instrument comprises 19 items categorized into seven components: subjective sleep quality, sleep latency, sleep duration, habitual sleep efficiency, sleep disturbances, sleep medication use, and daytime dysfunction. Individual component scores are aggregated to compute a global score ranging from 0 to 21; higher scores show poorer sleep quality. A total score of five or above suggests inadequate sleep quality. Rakhshani et al. [24] documented test-retest reliability of 0.78 and internal consistency (Cronbach's alpha) of 0.85 for the PSQI. This study revealed acceptable internal consistency of the PSQI, as indicated by a Cronbach's alpha of 0.77.

### 2.5. Data collection

Data collection was conducted using an electronic self-administered questionnaire delivered through the https://porsline.ir/ survey platform. Eligible ED nurses were informed about the study objectives and procedures through official communication channels of the participating hospitals. The survey link was disseminated via the ED`s authorized messaging groups on Telegram, WhatsApp, and Bale.

Participation was entirely voluntary, and electronic informed consent was obtained before participants could access the questionnaire. To optimize participation, two reminder messages were posted in the same official groups at one‑week intervals. The platform was configured to restrict each participant to a single submission by limiting repeat entries from the same device/IP address.

Because the distribution of the survey link occurred through existing ED messaging groups, the total number of nurses who received or viewed the invitation could not be determined. As a result, a traditional response rate could not be calculated. Instead, among nurses who accessed the survey and provided electronic consent, the completion rate was high, which reflects the mandatory items design rather than universal participation among all invited nurses.

Before the main data collection, a pilot pre‑survey was conducted with a convenience sample of 10 ED nurses from one of the participating hospitals. The pilot aimed to evaluate the clarity, comprehensibility, and length of the questionnaire, as well as the technical performance of the electronic survey on the platform. Participants were asked to provide feedback on any ambiguous wording, difficult items, or technical problems. Based on their comments, minor wording adjustments were made to improve clarity; no structural changes to the scales were required. All responses were exported from the Porsline system into Excel and subsequently imported into Stata version 16 for data cleaning and statistical analysis. Minor missing values within otherwise valid questionnaires were handled according to the scoring guidelines of each instrument; cases exceeding the missingness threshold were excluded from the final dataset.

### 2.6. Ethical consideration

Following ethical approval from the Shahid Beheshti University of Medical Sciences Ethics Research Center (IR.SBMU.RETECH.REC.1403.857) and hospital access authorization, each participant was provided a comprehensive explanation of the study's aims. Informed consent was obtained, and participant confidentiality was assured. For privacy reasons, participants were informed that providing personal identifiers, such as names or surnames, was not mandatory. Furthermore, participants received instructions on questionnaire completion and were informed of their voluntary participation. The corresponding author and the Medical Ethics and Law Research Center maintained the exclusive custody and management of all collected data, which are conditionally available to other researchers for non-commercial research purposes, following a request and subsequent approval.

### 2.7. Data analysis

Quantitative data were analyzed using descriptive statistics, including means and standard deviations for continuous variables and frequencies and percentages for categorical variables.

The normality of continuous variables was assessed using both graphical methods (Q–Q plots, P–P plots, and histograms) and statistical testing (Kolmogorov–Smirnov test). Given deviations from normality, non‑parametric tests were applied. Spearman’s rank correlation coefficient was used to examine associations between primary study variables and continuous demographic factors. For comparisons involving categorical demographic variables, the Mann–Whitney U test was employed for two-category variables, and the Kruskal–Wallis H test was used for variables with more than two categories.

To identify predictors of turnover intention, robust regression analysis was performed using Stata version 16 (StataCorp, College Station, TX, USA). M‑estimation through an iteratively reweighted least squares (IRLS) procedure was implemented. The algorithm uses Huber weighting in the initial stage and biweight (bisquare) weighting in subsequent iterations to reduce the influence of outliers and influential observations. This approach was selected due to the non‑normal distribution of residuals and the presence of influential cases identified during diagnostic assessment. To assess potential multicollinearity among predictors, particularly compassion satisfaction, burnout, and STS, we calculated variance inflation factors (VIFs). All variables demonstrated acceptable levels of multicollinearity (VIF < 5), meeting established diagnostic criteria.

A forced-entry (enter) robust regression model was used rather than stepwise selection. This approach was chosen because all predictors were conceptually important, supported by previous empirical research, and were identified a priori as relevant factors for turnover intention. Stepwise procedures were avoided because they may exclude important variables due to sample-specific fluctuations, and produce models with reduced stability and generalizability. A two‑tailed significance level of 0.05 was considered statistically significant.

## 3. Results

### 3.1. Participants’ description

180 ED nurses participate in the study. The participants had a mean age of 34.65 (SD = 7.06). Table 1 presents the demographic characteristics of the sample. The majority were female (n = 127, 70.6%). Additionally, 151 participants held a bachelor's degree, and 147 reported working rotating shifts. Regarding the nurse-to-patient ratio, 73.9% reported being responsible for a ratio of 1:5 or higher. Approximately half of the respondents (50.6%) described their financial status as good.

**Table 1:** Demographic characteristics of included participants (N = 180).

Mean ± SD or N (%)	Characteristics
34.65 ± 7.06	**Age (Year)**
16.81 ± 5.42	**Monthly Shifts (Day)**
8.82 ± 5.84	**Occupational Experiences (Year)**
**Gender**	
53 (29.4%)	Male
127 (70.6%)	Female
**Marital status**	
57 (31.7%)	Single
113 (62.8%)	Married
10 (5.6%)	Divorced
**Academic Level**	
151 (83.9%)	BSN
29 (16.1%)	MSN
**Financial Status**	
17 (9.4%)	Weak
72 (40.0%)	Moderate
91 (50.6%)	Good
**Shift Type**	
16 (8.9%)	Morning
13 (7.2%)	Night
147 (81.7%)	Rotation
4 (2.2%)	Two Shift Work
**Nurse-Patients Ratio**	
5 (2.8%)	1:2
25 (13.9%)	1:3
17 (9.4%)	1:4
133 (73.9%)	1:5 or higher

### 3.2. Major study variables

#### 3.2.1 Descriptive analysis.

The participants reported a mean turnover intention score of 49.26 (SD = 10.59). The average workplace stress level was 198.27 (SD = 34.52). The mean sleep quality score, as assessed by the Pittsburgh Sleep Quality Index (PSQI), was 8.59 (SD = 3.98), with 161 participants (89.4%) indicating poor sleep quality. The Pro-QOL mean scores were as follows: burnout, 30.30 (SD = 5.85); compassion satisfaction, 35.76 (SD = 6.49); and secondary traumatic stress (STS), 31.91 (SD = 6.78). A comprehensive summary of the key study variables is presented in Table 2.

**Table 2:** Descriptive statistics for Professional Quality of Life, Workplace Stress, Sleep Quality, and Turnover Intention among ED nurses.

Variable		Mean (SD)
**Turnover Intention**		49.26 (10.59)
**Workplace Stress**		198.27 (34.52)
**Sleep Quality**		8.59 (3.98)
**Sleep Quality**	**Poor**, n (%)	161 (89.4%)
	**Good**, n (%)	19 (10.6%)
**ProQoL**	**Burnout**	30.30 (5.85)
	**Compassion Satisfaction**	35.76 (6.49)
	**STS**	31.91 (6.78)

#### 3.2.2 Test of Normality Analysis.

The results of Kolmogorov-Smirnov and Shapiro-Wilk tests showed that the data for major study variables were not normally distributed (P < 0.005) (S1 Appendix).

#### 3.2.3 Relationships between turnover intention, workplace stress, sleep quality, compassion satisfaction, burnout, and STS.

As shown in Table 3, turnover intention was statistically significantly correlated with workplace stress (r = 0.408, p < 0.001), sleep quality (r = 0.329, p < 0.001) and all sub-dimensions of Pro-QoL including compassion satisfaction (r = −0.555, p < 0.001), burnout (r = 0.575, p < 0.001) and STS (r = 0.594, p < 0.001). Regarding other relationships, we couldn't find a significant relationship between workplace stress and burnout (r = 0.142, p = 0.057). Also, workplace stress and sleep quality were not significantly correlated (r = 0.137, p = 0.066). All other correlations were significant (p ≤ 0.05).

**Table 3:** Correlations of major study variables (N = 180).

Variable			CS	Burnout	STS	Turnover Intention	Workplace Stress	Sleep Quality
**Spearman's rho**	**CS**	**r**	1.000					
		**p-value**	.					
		**N**	180					
	**Burnout**	**r**	−0.518^**^	1.000				
		**p-value**	<0.001	.				
		**N**	180	180				
	**STS**	**r**	−0.406^**^	0.471^**^	1.000			
		**p-value**	<0.001	<0.001	.			
		**N**	180	180	180			
	**Turnover Intention**	**r**	−0.555	0.575	0.594	1.000		
		**p-value**	<0.001	<0.001	<0.001	.		
		**N**	180	180	180	180		
	**Workplace Stress**	**r**	−0.148	0.142	0.382	0.408	1.000	
		**p-value**	0.048	0.057	<0.001	<0.001	.	
		**N**	180	180	180	180	180	
	**Sleep Quality**	**r**	−0.250	0.396	0.353	0.329	0.137	1.000
		**p-value**	<0.001	<0.001	<0.001	<0.001	0.066	.
		**N**	180	180	180	180	180	180

#### 3.2.4 Demographic factors related to turnover intention.

Results showed that a master's degree was associated with lower turnover intention scores (p = 0.039). Also, better financial status was related to lower turnover intention scores (p < 0.001). A 1:3 nurse-to-patient ratio was significantly associated with higher turnover intention scores (p < 0.001). As the last significant factor, night shifts were associated with higher turnover intention scores (p < 0.001) (Table 4).

**Table 4:** Comparison of nurses’ turnover intention by sample characteristics (N = 180).

Charactristics	N	Mean	Std. Deviation	P value
**Gender**				
Female	127	49.46	10.17	0.640^a^
Male	53	48.77	11.62	
**Marital Status**				
Single	57	49.91	12.79	0.786^b^
Married	113	48.87	9.69	
Divorced	10	50.00	6.16	
**Education**				
BSN	151	49.85	10.45	0.037^a^
MSN	29	46.17	10.95	
**Financial Status**				
Weak	17	61.18	5.14	<0.001^b^
Moderate	72	49.08	10.82	
Good	91	47.18	9.74	
**N:P Ratio**				
1:2	5	60.80	4.38	<0.001^b^
1:3	25	61.24	13.43	
1:4	17	51.41	8.60	
1:5 or higher	133	46.30	8.25	
**Shift Type**				
Morning	16	48.00	9.73	<0.001^b^
Night	13	64.08	6.56	
Rotation	147	47.69	9.79	
Two Shift	4	59.00	10.83	

a Mann-Whitney U-Test.

b Kruskal-Wallis test.

#### 3.2.5 Predictors of turnover intention among ED nurses.

Table 5 presents the results of robust regression analysis, which examined the predictors of ED nurses’ turnover intention. First, all major study variables were entered into the model. In this model, all factors significantly contributed to turnover intention (p ≤ 0.05). In the second model, all statistically significant demographic factors were added to the first model. In this model, compassion satisfaction, burnout, STS, job stress, moderate financial status, good financial status, 1:3 nurse-to-patient ratio, and rotation shift type significantly contributed to turnover intention (p ≤ .05).

**Table 5:** Results of robust regression analysis.

Dependent variable: Turnover intention total score								
	Model 1				Model 2			
**Variables**	**B**	**P**	**95.0% CI for B**		**B**	**P**	**95.0% CI for B**	
			**Lower**	**Upper**			**Lower**	**Upper**
STS	0.81	**<0.001**	0.65	0.97	0.65	**<0.001**	0.47	0.83
Burnout	0.22	**0.021**	0.03	0.40	0.29	**0.006**	0.08	0.49
Compassion Satisfaction	−0.33	**<0.001**	−0.47	−0.18	−0.38	**<0.001**	−0.54	−0.22
Workplace Stress	0.06	**<0.001**	0.03	0.08	0.05	**<0.001**	0.02	0.09
Sleep Quality	0.28	**0.021**	0.04	0.51	−0.19	0.182	−0.47	0.09
Education (MSN)	–	–	–	–	−0.28	0.810	−2.61	2.04
Financial Status (Moderate)	–	–	–	–	−6.86	**<0.001**	−10.40	−3.33
Financial Status (Good)	–	–	–	–	−8.33	**<0.001**	−12.06	−4.60
N:P Ratio (1:3)	–	–	–	–	7.06	**0.019**	1.18	12.95
N:P Ratio (1:4)	–	–	–	–	0.27	0.930	−5.89	6.44
N:P Ratio (1:5)	–	–	–	–	1.14	0.696	−4.61	6.89
Shift Type (Night)	–	–	–	–	3.14	0.218	−1.88	8.16
Shift Type (Rotation)	–	–	–	–	3.23	**0.049**	0.01	6.45
Shift Type (Two Shift Work)	–	–	–	–	0.50	0.885	−6.29	7.29
Constant	13.95	0.05	4.37	23.53	26.01	**<0.001**	12.60	39.42

**Note:** The table presents unstandardized coefficients (B), p-values (P), and 95% confidence intervals (CI) for the coefficients; Reference categories: Education = *Bachelor’s degree (BSN)*; Financial status = *poor*; Nurse-to-patient ratio = *1:2*; Shift type = *Morning shift*.

## 4. Discussion

This cross-sectional study examined the relationships among Pro-QoL, sleep quality, occupational stress, and turnover intention in 180 ED nurses across hospitals in Tehran, Iran. The principal finding is that all major variables, with the exception of occupational stress with burnout, and occupational stress with sleep quality, were significantly correlated with turnover intention. In robust regression, compassion satisfaction, burnout, STS, job stress, financial status, nurse-to-patient ratio, and shift type were the significant independent predictors.

Findings from a cross-sectional study conducted in China in 2022 indicated that health status, frequency of monthly night shifts, and factors related to the work environment were all significantly correlated with the turnover intention [25]. A study in Ethiopia in 2020 found that more than 77% of ED nurses intended to leave their current workplace. Factors that significantly influenced the turnover intention of nurses included education, monthly income, and autonomy [26]. In our study, financial status also impacted the turnover intention significantly. In this respect, our study is similar to the study mentioned above.

Although the mean ProQOL subscale scores of the present sample fell within the moderate band of the ProQOL manual, these values should not be read as reassuring in this setting. The same nurses recorded a mean PSQI score of 8.59, with 89.4% classified as poor sleepers, and a mean turnover intention score of 49.26. Moderate burnout and STS therefore co-occurred with an almost universal sleep disturbance and with turnover intention scores that were significantly correlated with burnout and STS. Moderate ProQOL scores in ED nurses may thus be clinically concerning rather than benign, because they mark a level at which compassion fatigue already coexists with measurable sleep impairment and a stated intention to leave. Given the cross-sectional design, this interpretation describes co-occurrence and not causation.

In China, Yang Ma et al. (2021) found that job stress, burnout, and job satisfaction were key factors associated with turnover intention among ED nurses [27]. These results are consistent with our study regarding the correlation between job stress, burnout, and compassion satisfaction. Another study by Lee et al. showed a significant relationship between turnover intention and burnout [28]. In this study, burnout was operationalized via the ProQOL burnout subscale, and similarly demonstrated a significant association with turnover intention, extending these findings to the Iranian ED context. It was found that job satisfaction was significantly related to intention to leave the job and was associated with lower intention to leave the job [29]. In our study, it was found that compassion satisfaction was significantly related to intention to leave the job. Our results are consistent with the study mentioned above.

A scoping review study aimed at assessing factors influencing intention to leave the profession showed that key factors influencing the resignation of ED nurses included workplace violence, burnout, depression, organizational characteristics such as poor quality of information provision in the organization, and lack of nurse/physician cooperation. Other key factors influencing resignation were environmental and job characteristics such as unmanageable work demands and pressures, and lack of job engagement [30]. In our study, as in the aforementioned review study, it was found that burnout, workplace stress, Pro-QoL, type of shifts, and nurse-to-patient significantly affect the intention to leave the service of emergency nurses.

In the present study, a weak positive correlation was observed between sleep quality and job stress (Spearman’s ρ = 0.137, P = 0.066). This finding is consistent with a previous study among nurses that also reported a positive association between these variables (r = 0.176, P = 0.036). However, unlike that study, the association in the present research did not reach statistical significance. This discrepancy may partly be explained by differences in the statistical methods used, including the application of Spearman’s rank correlation in the current study instead of Pearson’s correlation, as well as potential differences in sample characteristics or data distribution [31]. Also, other mechanisms may explain these null findings. First, the sample was highly homogeneous in terms of sleep quality, 89.4% reported poor sleep, suggesting a potential ceiling effect that would attenuate the correlation. Second, the Nursing Stress Scale captures multiple dimensions of occupational stress, some of which may be less directly linked to sleep disruption than shift-related physiological fatigue. Third, instrument timing relative to shift schedules was not controlled; measurements taken close to or following night shifts may not accurately reflect habitual sleep quality. Future longitudinal studies with repeated measures and larger samples are needed to clarify these relationships.

Nurses assigned a 1:3 ratio reported markedly higher turnover intention scores than those with a 1:5 or higher ratio. This finding is counterintuitive in the context of the workload-stress-attrition literature, which generally predicts that higher patient loads generate more stress and greater attrition. Several explanations merit consideration. In tertiary ED settings, nurses assigned lower ratios (1:3) may be responsible for managing the most critically ill or hemodynamically unstable patients, resulting in disproportionately high psychological burden despite a numerically lower patient count. Alternatively, assignment to a 1:3 ratio may reflect a particular sub-specialty (e.g., resuscitation bays) with distinct stressors not captured by global workload measures. Confounding by shift type, seniority, or ward acuity is also possible. This finding should be interpreted cautiously and warrants prospective investigation with detailed workload characterization.

A study found that the overall prevalence of turnover intention among emergency nurses was 45%. Subsequent analysis indicated a higher prevalence in Asia at 54%, compared to 38% in other regions [32]. The findings of this study highlight the significance of identifying the underlying factors contributing to the high prevalence of turnover intention among ED nurses, particularly in Asian countries. Several of these factors have been explored in the present study and may serve as a foundation for future research to develop management-focused interventions to reduce nurse turnover rates, especially among emergency nursing staff. Nursing staff shortages in Iran, which have intensified workloads and contributed to higher levels of burnout, are well-documented [33]. These findings highlight the importance of implementing targeted institutional strategies, including reforming financial incentives, ensuring equitable shift scheduling, and providing structured psychosocial support for ED nurses.

### 4.1 Limitation

First, the sample’s representativeness is limited, as the study was conducted on a relatively small and specific population of emergency department nurses. Second, due to the study’s cross‑sectional design, only associations can be inferred rather than definitive causal relationships; therefore, future longitudinal designs are recommended to examine causal pathways more precisely. Third, the use of a convenience sampling approach and online recruitment may have introduced selection bias, as nurses who were more active in digital communication groups or more interested in the study topic might have been more likely to participate. This potential bias should be considered when generalizing the findings to broader nursing populations.

## 5. Conclusions

This cross-sectional study found that Pro-QoL, specifically burnout, STS, and compassion satisfaction, occupational stress, and sleep quality were all significantly associated with turnover intention among ED nurses, except for the occupational stress–burnout and occupational stress–sleep quality dyads. Regression analysis identified burnout, STS, compassion satisfaction, job stress, poor financial status, a 1:3 nurse-to-patient ratio, and rotating shift type as significant predictors of turnover intention. These associations highlight priority domains for organizational intervention, including financial compensation, shift scheduling reform, and support for professional well-being. However, given the cross-sectional design, these findings reflect associations only; longitudinal and interventional research is required to determine whether addressing these factors effectively reduces nurse turnover in ED settings.

## Supporting information

S1 Appendix Test of normality results. (DOCX)

## References

[1] Młynarska A, Bronder M, Kolarczyk E, Manulik S, Młynarski R. Determinants of sleep disorders and occupational burnout among nurses: a cross-sectional study. Int J Environ Res Public Health. 2022;19(10):6218. doi: 10.3390/ijerph19106218 35627754 PMC9140934

[2] Wong CL, Young B, Lui BSC, Leung AWY, So JLT. Professional quality of life and resilience in emergency department healthcare professionals during COVID-19 in Hong Kong: a cross-sectional study. Hong Kong Journal of Emergency Medicine. 2022;29(3):168–76.

[3] Ayed A, Abu Ejheisheh M, Aqtam I, Batran A, Farajallah M. The relationship between professional quality of life and work environment among nurses in intensive care units. Inquiry. 2024;61:469580241297974. doi: 10.1177/00469580241297974 39520216 PMC11550503

[4] Petrosino F, Bartoli D, Trotta F, Di Nome S, Di Sarli MG, Frammartino R, et al. Nurses quality of life, sleep disturbance, and intention to leave critical care units: a cross-sectional moderated mediation analysis. Intensive Crit Care Nurs. 2024;81:103602. doi: 10.1016/j.iccn.2023.103602 38101214

[5] Buselli R, Corsi M, Baldanzi S, Chiumiento M, Del Lupo E, Dell’Oste V, et al. Professional quality of life and mental health outcomes among health care workers exposed to Sars-Cov-2 (Covid-19). Int J Environ Res Public Health. 2020;17(17):6180. doi: 10.3390/ijerph17176180 32858810 PMC7504107

[6] Stamm B. The concise manual for the professional quality of life scale; 2010.

[7] Ganesan S, Magee M, Stone JE, Mulhall MD, Collins A, Howard ME, et al. The impact of shift work on sleep, alertness and performance in healthcare workers. Sci Rep. 2019;9(1):4635. doi: 10.1038/s41598-019-40914-x 30874565 PMC6420632

[8] Dong H, Zhang Q, Zhu C, Lv Q. Sleep quality of nurses in the emergency department of public hospitals in China and its influencing factors: a cross-sectional study. Health Qual Life Outcomes. 2020;18(1):116. doi: 10.1186/s12955-020-01374-4 32349759 PMC7191763

[9] Alrasheeday A, Alsaeed MA, Alshammari B, Alshammari F, Alrashidi AS, Alsaif TA, et al. Sleep quality among emergency nurses and its influencing factors during COVID-19 pandemic: a cross-sectional study. Front Psychol. 2024;15:1363527. doi: 10.3389/fpsyg.2024.1363527 39100564 PMC11297352

[10] Ebrahimian A, Fakhr-Movahedi A, Hashemi-Amrei SH. The relationship between the emergency nurses’ sleep quality and the sleep quality of their spouses: a cross-sectional descriptive-analytical study. Health Sci Rep. 2023;6(1):e965. 10.1002/hsr2.965 PMC9709222 36467759

[11] Li N, Zhang L, Xiao G, Chen ZJ, Lu Q. Effects of organizational commitment, job satisfaction and workplace violence on turnover intention of emergency nurses: A cross-sectional study. Int J Nurs Pract. 2020;26(6):e12854. doi: 10.1111/ijn.12854 32529786

[12] Mafula D, Arifin H, Chen R, Sung C-M, Lee C-K, Chiang K-J, et al. Prevalence and moderating factors of turnover rate and turnover intention among nurses worldwide: a meta-analysis. J Nurs Regul. 2025;15(4):20–36. doi: 10.1016/s2155-8256(25)00031-6

[13] Ren H, Xue Y, Li P, Yin X, Xin W, Li H. Prevalence of turnover intention among emergency nurses worldwide: a meta-analysis. BMC Nurs. 2024;23(1):645. doi: 10.1186/s12912-024-02284-2 39261866 PMC11389441

[14] Wubetie A, Taye B, Girma B. Magnitude of turnover intention and associated factors among nurses working in emergency departments of governmental hospitals in Addis Ababa, Ethiopia: a cross-sectional institutional based study. BMC Nurs. 2020;19:97. doi: 10.1186/s12912-020-00490-2 33071646 PMC7556577

[15] von Elm E, Altman DG, Egger M, Pocock SJ, Gøtzsche PC, Vandenbroucke JP, et al. The Strengthening the Reporting of Observational Studies in Epidemiology (STROBE) statement: guidelines for reporting observational studies. J Clin Epidemiol. 2008;61(4):344–9. doi: 10.1016/j.jclinepi.2007.11.008 18313558

[16] Lee Y-W, Dai Y-T, McCreary LL. Quality of work life as a predictor of nurses’ intention to leave units, organisations and the profession. J Nurs Manag. 2015;23(4):521–31. doi: 10.1111/jonm.12166 24238014

[17] Barzanji KH. Comparison of the tendency to leave the service in government employees Payam Noor University; 2013.

[18] Mirzaei A, Rezakhani Moghaddam H, Habibi Soola A. Identifying the predictors of turnover intention based on psychosocial factors of nurses during the COVID-19 outbreak. Nurs Open. 2021;8(6):3469–76. doi: 10.1002/nop2.896 33960721 PMC8242757

[19] Heidari A, Kazemi SB, Kabir MJ, Khatirnamani Z, Badakhshan A, Lotfi M, et al. Relationship between turnover intention and perceived organizational support in nurses of hospitals in Gorgan, Iran. J Mod Med Inf Sci. 2023;9(3):234–45.

[20] Ghorji M, Keshavarz Z, Ebadi A, Nasiri M. Persian translation and psychometric properties of Professional Quality of Life Scale (ProQOL) for health care providers. J Mazandaran Univ Med Sci. 2018;28:93–106.

[21] Gray-Toft P, Anderson JG. The nursing stress scale: development of an instrument. J Behav Assess. 1981;3(1):11–23. doi: 10.1007/bf01321348

[22] Olyaie KR, Haghani S, Khayeri F, Seyedfatemi N. Evaluating the effect of positive self-talk on job stress among nurses working in the emergency wards; 2020.

[23] Buysse DJ, Reynolds CF 3rd, Monk TH, Berman SR, Kupfer DJ. The Pittsburgh Sleep Quality Index: a new instrument for psychiatric practice and research. Psychiatry Res. 1989;28(2):193–213. doi: 10.1016/0165-1781(89)90047-4 2748771

[24] Rakhshani M, Akbarzadeh R, Koshan M, Hashemi Nik SM. Effect of the benson relaxation technique on quality of sleep in patients with chronic heart disease. J Sabzevar Univ Med Sci. 2014;21(3):492–50.

[25] Jiang N, Zhou X, Gong Y, Tian M, Wu Y, Zhang J, et al. Factors related to turnover intention among emergency department nurses in China: a nationwide cross-sectional study. Nurs Crit Care. 2023;28(2):236–44. doi: 10.1111/nicc.12770 35384173

[26] Wubetie A, Taye B, Girma B. Magnitude of turnover intention and associated factors among nurses working in emergency departments of governmental hospitals in Addis Ababa, Ethiopia: a cross-sectional institutional based study. BMC Nurs. 2020;19:97. doi: 10.1186/s12912-020-00490-2 33071646 PMC7556577

[27] Ma Y, Chen F, Xing D, Meng Q, Zhang Y. Study on the associated factors of turnover intention among emergency nurses in China and the relationship between major factors. Int Emerg Nurs. 2022;60:101106. doi: 10.1016/j.ienj.2021.101106 34864323

[28] Lee MMD, Gensimore MM, Maduro RS, Morgan MK, Zimbro KS. The impact of burnout on emergency nurses’ intent to leave: a cross-sectional survey. J Emerg Nurs. 2021;47(6):892–901. doi: 10.1016/j.jen.2021.07.004 34417028

[29] Li N, Zhang L, Xiao G, Chen J, Lu Q. The relationship between workplace violence, job satisfaction and turnover intention in emergency nurses. Int Emerg Nurs. 2019;45:50–5. doi: 10.1016/j.ienj.2019.02.001 30797732

[30] McIntyre N, Crilly J, Elder E. Factors that contribute to turnover and retention amongst emergency department nurses: A scoping review. Int Emerg Nurs. 2024;74:101437. doi: 10.1016/j.ienj.2024.101437 38583300

[31] Fadae Aghdam N, Ameri M, Goli S, Imeni M. Relationship between sleep quality and job stress of nurses in different shifts working. Avicenna J Nurs Midwifery care. 2020;28(2):103–11. doi: 10.30699/ajnmc.28.2.103

[32] Ren H, Xue Y, Li P, Yin X, Xin W, Li H. Prevalence of turnover intention among emergency nurses worldwide: a meta-analysis. BMC Nurs. 2024;23(1):645. doi: 10.1186/s12912-024-02284-2 39261866 PMC11389441

[33] Hajizadeh A, Kakemam E, Arab-Zozani M, Najafpoor Z, Dargahi H, Saeidpour J. Turnover intention among nurses in Iranian hospitals: a systematic review and meta-analysis. BMC Nurs. 2025;24(1):166. doi: 10.1186/s12912-025-02801-x 39948560 PMC11827215

