# Supplementary material for: Professional quality of life, sleep quality, occupational stress, and turnover intention among emergency department nurses: A cross-sectional study
Source: PLoS One. 2026 Sep 22;21(9):e0357570. doi: 10.1371/journal.pone.0357570 (PMC13596796; doi:10.1371/journal.pone.0357570)
Supplement: Supplementary file 1 [file pone.0357570.s001.docx]

| **Variable** | **Kolmogorov-Smirnov^a^** | | | **Shapiro-Wilk** | | |
| --- | --- | --- | --- | --- | --- | --- |
|  | **Statistic** | **df** | **Sig.** | **Statistic** | **df** | **Sig.** |
| **Turnover Intention** | .160 | 179 | .000 | .950 | 179 | .000 |
| **Compassion Satisfaction** | .084 | 179 | .004 | .981 | 179 | .016 |
| **Burnout** | .151 | 179 | .000 | .940 | 179 | .000 |
| **STS** | .167 | 179 | .000 | .935 | 179 | .000 |
| **Workplace Stress** | .089 | 179 | .002 | .967 | 179 | .000 |
| **Sleep Quality** | .201 | 179 | .000 | .907 | 179 | .000 |

**S1 Appendix.** Test of normality results.
